# Copper Nanoparticle-Incorporated Carbon Fibers as Free-Standing Anodes for Lithium-Ion Batteries

**DOI:** 10.1186/s11671-016-1389-6

**Published:** 2016-03-31

**Authors:** Pan Han, Tao Yuan, Long Yao, Zhuo Han, Junhe Yang, Shiyou Zheng

**Affiliations:** School of Materials Science and Engineering, University of Shanghai for Science and Technology, Shanghai, 200093 China; School of Environment and Architecture, University of Shanghai for Science and Technology, Shanghai, 200093 China

**Keywords:** Electrospun, Carbon-fibers, Incorporation, Anode, Lithium-ion battery

## Abstract

Copper-incorporated carbon fibers (Cu/CF) as free-standing anodes for lithium-ion batteries are prepared by electrospinning technique following with calcination at 600, 700, and 800 °C. The structural properties of materials are characterized by X-ray diffraction (XRD), Raman, thermogravimetry (TGA), scanning electron microscopy (SEM), transmission electron microscope (TEM), and energy dispersive X-ray spectrometry (EDS). It is found that the Cu/CF composites have smooth, regular, and long fibrous morphologies with Cu nanoparticles uniformly dispersed in the carbon fibers. As free-standing anodes, the unique structural Cu/CF composites show stable and high reversible capacities, together with remarkable rate and cycling capabilities in Li-ion batteries. The Cu/CF calcined at 800 °C (Cu/CF-800) has the highest charge/discharge capacities, long-term stable cycling performance, and excellent rate performance; for instance, the Cu/CF-800 anode shows reversible charge/discharge capacities of around 800 mAh g^−1^ at a current density of 100 mA g^−1^ with stable cycling performance for more than 250 cycles; even when the current density increases to 2 A g^−1^, the Cu/CF-800 anode can still deliver a capacity of 300 mAh g^−1^. This excellent electrochemical performance is attributed to the special 1D structure of Cu/CF composites, the enhanced electrical conductivity, and more Li^+^ active positions by Cu nanoinclusion.

**Electronic supplementary material:**

The online version of this article (doi:10.1186/s11671-016-1389-6) contains supplementary material, which is available to authorized users.

## Background

Rechargeable lithium-ion batteries are used extensively due to their high energy and power densities [1–3]. State-of-the-art lithium secondary batteries are composed of graphite anode, which has low theoretical specific capacity (372 mAh g^−1^) and limited rate capability [4]. Thus, new carbon-based anode materials such as carbon nanotube [5], nanofiber [6], nanobead [7], hollow nanosphere [8], graphene [9], and their hybrids [10] with enhanced Li^+^ storage capacities and high rate performance have been explored as alternative candidates for anode of Li-ion batteries. Among them, one-dimensional (1D)-structured materials such as fibers, rods, and nanotubes can usually improve the physical or chemical interactions of the electrodes with lithium ions since their large surface-to-volume ratio and fast electronic conducting pathway.

Furthermore, with the recent improvements in lightweight and flexible battery for potential applications in portable and bendable electronic equipment, for example, wearable devices, implantable medical devices, distributed sensors, and soft free-standing electrode-active materials without binder and conductive agent are significantly for such flexible batteries. Many advanced techniques have been developed to fabricate flexible free-standing carbonous electrodes, for instance, vacuum filtration [11, 12], aerosol pyrolysis [13, 14], anodic oxidation [15], chemical vapor deposition [16], sol-gel deposition [17–19], sputtering [20], and spreading [21]. Electrospinning also turns out to be a simple and versatile method for generating ultrathin fibers and hollow fibers [22–26]. Several researchers have successfully applied the electrospinning technique for the fabrication of non-woven film electrodes in lithium-ion batteries [26–29].

In the present work, we prepared copper-incorporated carbon fibers (Cu/CF) by electrospinning Cu(NO_3_)_2_ and polyacrylonitrile (PAN)-mixed solution and subsequent thermal treatment at different temperatures. The structural and electrochemical properties of the flexible non-woven Cu/CF films were systematically investigated. The Cu/CF composites show smooth, regular, and long fibrous morphologies with Cu nanoparticles uniformly dispersed in the carbon fibers. The Cu/CF sample annealed at 800 °C (Cu/CF-800) shows higher charge/discharge capacities and long-term stable cycling performance (250 deep charge-discharge cycles) under the current density of 100 mA g^−1^ and excellent rate performance, which is attributed to 1D continues cross-link structure of the film, together with increased electrical conductivity and active position for Li^+^ intercalation/de-intercalation with Cu nanoparticles implanted into fibers.

## Methods

### Preparation of Cu/CF Composites

Polyacrylonitrile (PAN, MW = 150000 g mol^−1^, Scientific Polymer Products) and *N*,*N*-dimethylformamide (DMF, 99 %) were purchased and used as received from Sigma-Aldrich. Copper dinitrate, Cu(NO_3_)_2_·3H_2_O (Aldrich), was used as the copper precursor.

First, 1 g of PAN was added into 10 mL of DMF to form a homogeneous and transparent polymeric solution after it was vigorously stirred for 3 h. Subsequently, 1 g of the Cu(NO_3_)_2_·3H_2_O was dissolved in above polymeric PAN solution. This solution was continuously stirred for 24 h at room temperature conditions leading to the formation of pale blue-colored copper hydroxide/PAN sol. The as-prepared sol was transferred to 10 mL syringe with a hypodermic needle (diameter 27 G) in a controlled electrospinning setup. The electrospinning process was then carried out with a high voltage (18 kV) at a flow rate of 0.5 mL h^−1^. A white, ultrafine membrane consisting of fibers could be collected on the alumina foil 15 cm away from the needle tip. The fibrous mat was further dried in the oven at 80 °C to evaporate all DMF solvent.

The as-prepared electrospun fibers were first stabilized in an ambient pressure at 280 °C for 2 h at a ramping rate of 2 °C min^−1^ and then carbonized at 600, 700, and 800 °C for 2 h under the protection of argon atmosphere, respectively. The corresponding products obtained were noted as Cu/CF-600, Cu/CF-700, and Cu/CF-800, respectively. For comparison, we also obtained bare CF-600, CF-700, and CF-800, not adding Cu(NO_3_)_2_·3H_2_O in the same conditions. To obtain the actual copper content in the Cu/CF samples, TGA was performed with a heating rate of 10 °C min^−1^ and highly pure N_2_ as the purge gas.

### Structural Characterization

XRD patterns were recorded on Rigaku D/max 2400, Japan, with Cu Kα radiation in the 2-theta range from 10°–80°. Raman spectra were scanned from 2850 to 100 cm^−1^ on a high-resolution dispersive Raman spectroscopic microscope (Horiba Jobin Yvon, USA). Scanning electron microscopy (SEM) images were obtained on a Hitachi S-4700, Japan, operating at 15 kV and equipped with an EDAX lithium-drifted silicon X-ray energy-dispersive spectrometer (XEDS). The transmission electron microscope (TEM) samples were examined in a JEOL (Japan) 2100F field emission TEM equipped with an energy dispersive X-ray spectrometry (EDS).

### Electrochemical Measurements

The non-woven Cu/CF films were cut into several wafers with a diameter of 14 mm as electrodes directly. Then, the Cu/CF electrodes were dried under vacuum at 100 °C for 12 h. Lithium metal foil (Kyokuto metal Co., Japan) as a counter electrode, 1 M LiPF_6_ in ethylene carbonate (EC), diethylcarbonate (DEC) (1:1 in volume) (Merck) as an electrolyte, and Celgard 2502 membrane as separator were assembled together with testing electrodes to obtain 2032-type coin cells in an argon-filled glove box (MBRAUN, Germany). Before all electrochemical measurements, cells were aged for 12 h and then tested for cyclic voltammetry (CV) measurement, charge-discharge cycling, rate performance, and electrochemical impedance spectra (EIS) studies. The charge and discharge performances of the batteries were tested with using LAND CT2001A battery test instrument (LAND Electronic Co., China), and potential ranges were controlled between 0.005 and 3 V (vs. Li/Li^+^) at ambient temperature. The specific capacity was calculated on the basis of the total quality of Cu/CF. The cyclic voltammetry (CV) measurement was conducted with a Gamry Reference 3000 (Gamry Co., USA) at a scan rate of 0.05 mV s^−1^. EIS was measured on the cell with a Gamry Reference 3000 at room temperature. The frequency ranged from 100 kHz to 100 mHz.

## Results and Discussion

### Morphology and Characterization

**Fig. 1:**
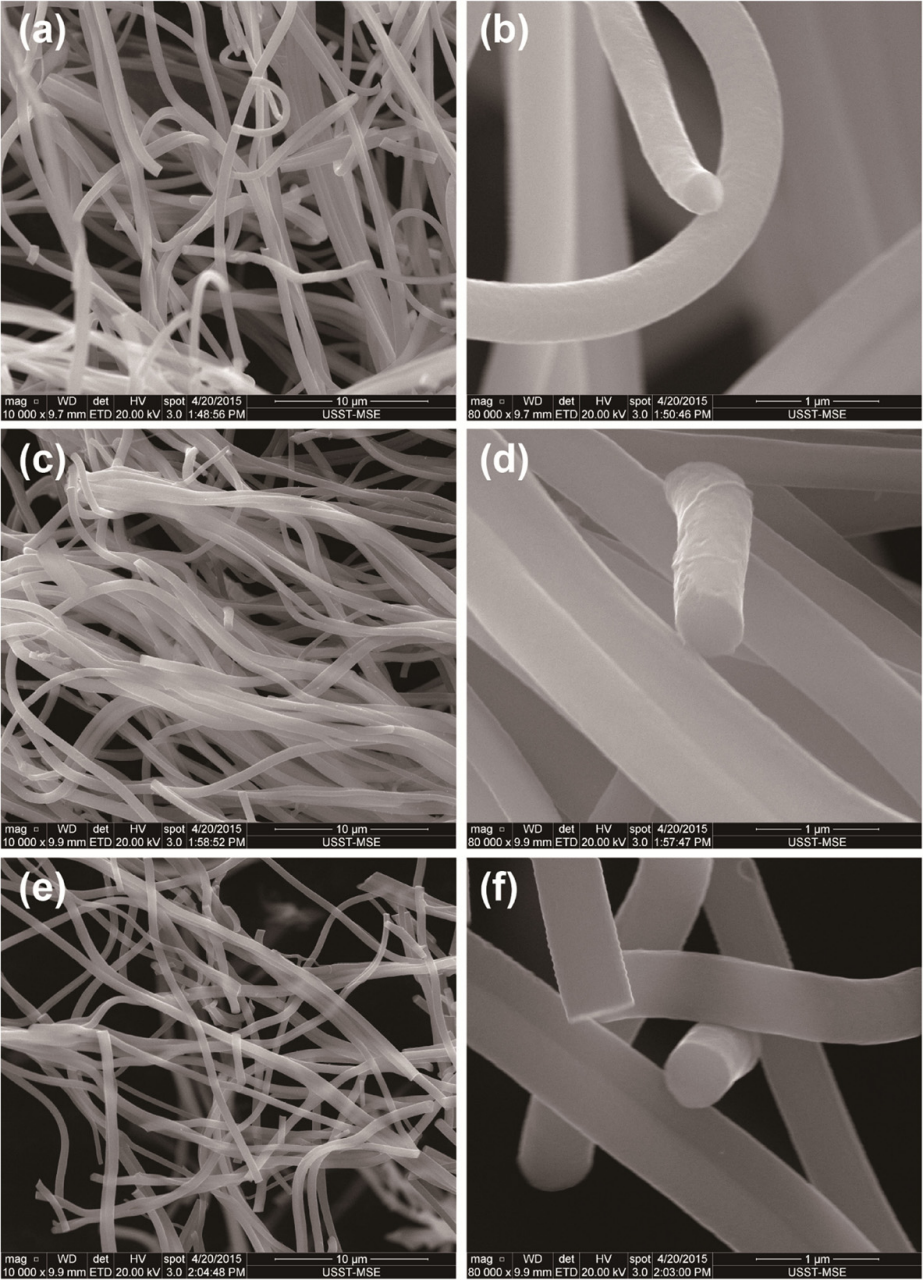
The SEM images of the carbonized composites at various temperatures in argon atmosphere. **a**, **b** Cu/CF-600. **c**, **d** Cu/CF-700. **e**, **f** Cu/CF-800

The morphologies of electrospinning Cu(NO_3_)_2_/PAN precursor fibers without calcination are shown in Additional file 1: Figure S1. The pristine Cu(NO_3_)_2_/PAN precursor fibers exhibit relatively smooth, long, and regular homogeneous diameter (~1 μm) morphologies, indicating a smooth injection of Cu(NO_3_)_2_/PAN precursor dispersed homogenously in the polymer matrix during the electrospinning process. The as-prepared non-woven films were then calcined at 600–800 °C in argon atmosphere for 2 h, to carbonize the organic substance and obtain Cu implanted carbon films. Figure 1 shows the SEM images of Cu/CF samples. After annealing, the straight fibrous morphology and netlike structure of as-obtained Cu/CF films were maintained from Cu(NO_3_)_2_/PAN precursor (Additional file 1: Figure S1). This 1D structure, along with the interconnecting nature can facilitate both fast electronic and ionic transport. In addition, the carbonized Cu/CF fibers have reduced average diameter (~500 nm), which may be ascribed to the complex chemical reactions (such as dehydration, dehydrogenation, and cyclization) during the process of carbonization to compare with the Cu(NO_3_)_2_/PAN precursor. As shown in Fig. 1, the average diameters of Cu/CF-600, Cu/CF-700, and Cu/CF-800 samples are no obvious different. All the obtained Cu/CF samples exhibit smooth surface and no Cu nanoparticles were found on the surface of the fibers, suggesting all the Cu particles were implanted in the carbon fibers, which can be further proved by TEM and corresponding energy dispersive X-ray spectrometry (EDS) mapping results.

**Fig. 2:**
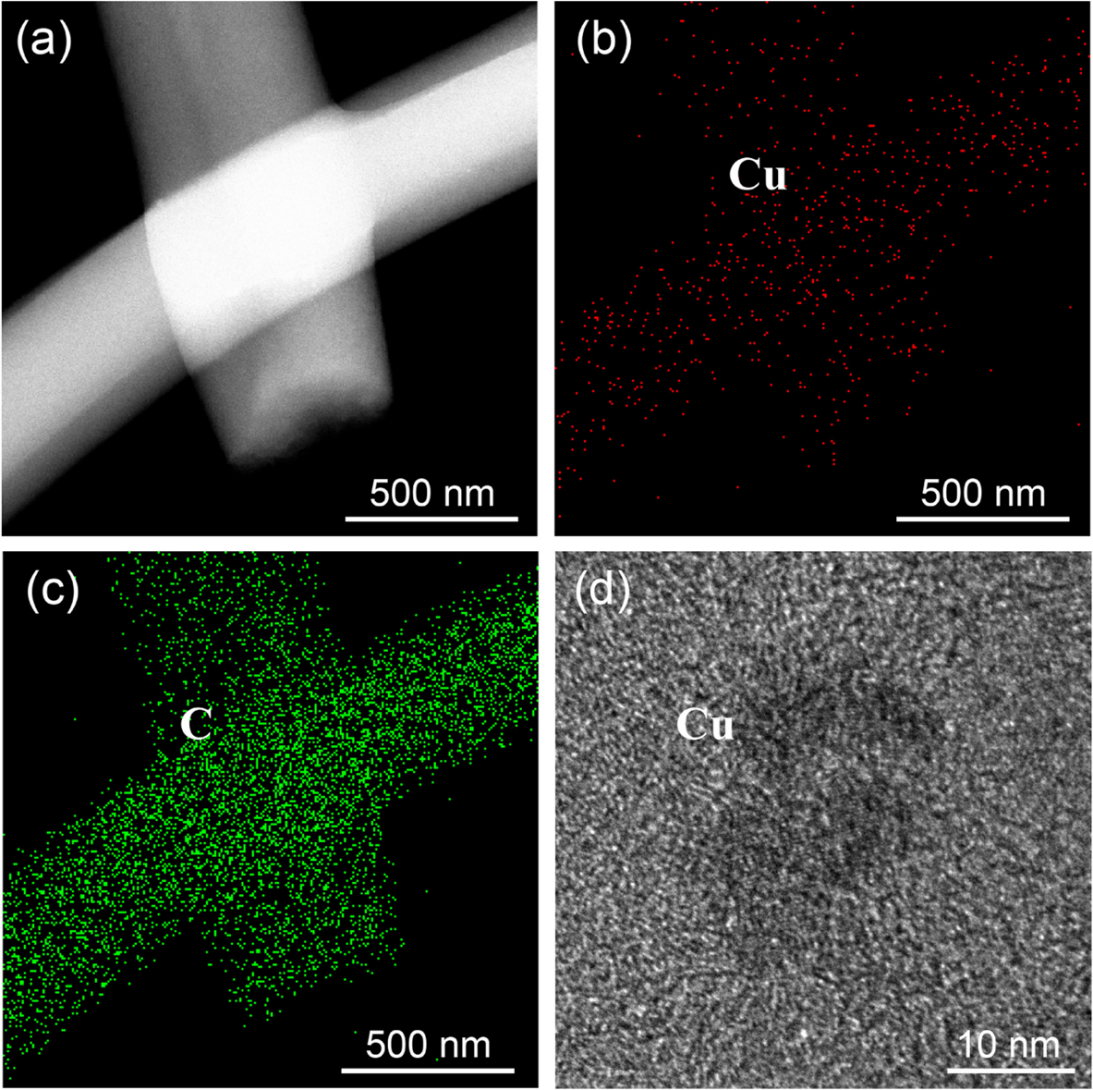
**a** TEM images of Cu/CF-800 composite and the corresponding EDS mapping of **b** C and **c** Cu and **d** HR-TEM image of Cu/CF-800 composite

Figure 2 shows the TEM images of Cu/CF-800 composites and the corresponding C and Cu EDS maps (Fig. 2b, c). The EDS intensity spectra illustrate a relatively quantitative ratio of elemental copper and carbon in the selected area for the Cu/CF-800 sample. In particular, the elemental mapping images of Cu and C show that the Cu maps cover the C maps. This clearly indicates that the Cu is well dispersed in the Cu/CF-800 composites fabricated by in situ electrospun technique. HR-TEM image of Cu/CF-800 composite (Fig. 2d) shows that Cu nanoparticles with a size of ~10 nm are distributed in the carbon matrix.

**Fig. 3:**
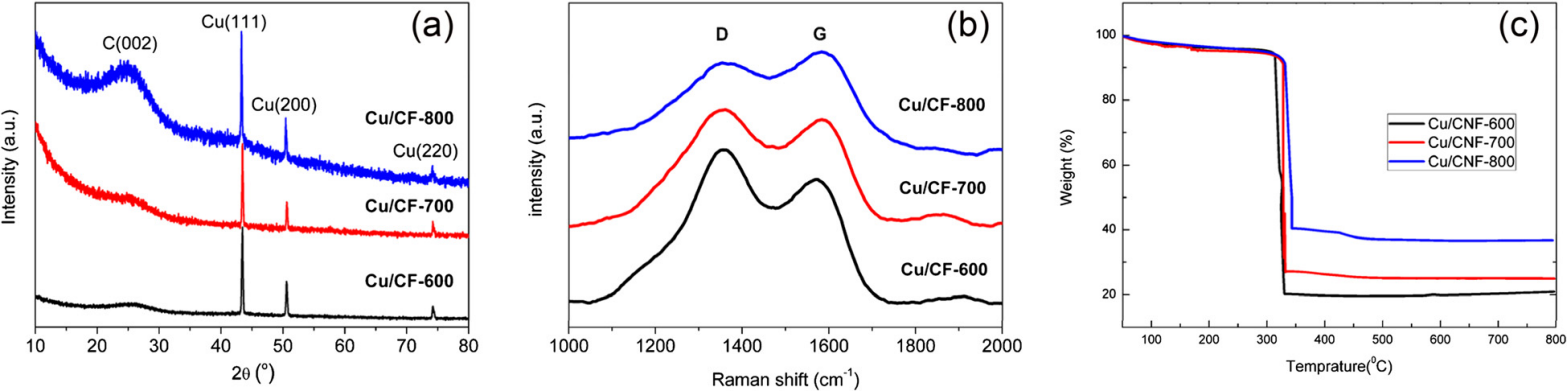
**a** XRD pattern, **b** Raman spectra, and **c** TGA curves for Cu/CF samples

X-ray diffraction patterns of electrospun Cu/CF samples are shown in Fig. 3a. The Bragg reflection at 2*θ* = 25° corresponding to the (002) plane shows a broad width, which suggests that all the as-obtained Cu/CF samples possess low degree of graphitization [30]. However, compare with Cu/CF-600 and Cu/CF-700 samples, the (002) peak intensity of Cu/CF-800 is stronger and higher, suggesting that better crystallinity of the carbon matrix was formed as the carbonization temperature increases to 800 °C [31, 32]. The crystallization peaks observed at 43.6°, 50.5°, and 74.5° of three Cu/CF samples correspond to the (111), (200), and (220) planes of fcc crystal structures of metallic Cu (JCPDS04-0836) [33, 34], indicating Cu nanoparticles well distributed within the carbon fibers.

Raman spectroscopy is a powerful and widely used technique for characterization of graphitization [35]. As shown in Fig. 3b, Raman spectra reveal the graphitization of Cu/CF treated under different carbonization temperature. All the samples display two prominent peaks, centered at about 1350 and 1600 cm^−1^, which correspond to the well-documented G band (E_2g_ mode of graphite) and D band (defect-induced mode). The intensity ratio of I_D_/I_G_ of Cu/CF-600, Cu/CF-700, and Cu/CF-800 is calculated to be 1.17, 1.00, and 0.91, respectively, which indicates that the degree of graphitization is increased with the increase of carbonization temperature. Based on the TGA result shown in Fig. 3c, the weight content of Cu in Cu/CF-800 is about 29.4 %, which is higher than Cu/CF-700 (19.9 %) and Cu/CF-600 (16.6 %). Combine with the above results, it is found that the carbonation temperature of 800 °C eliminates the residue organic of Cu(NO_3_)_2_/PAN precursor more efficiently.

### Electrochemical Measurement

**Fig. 4:**
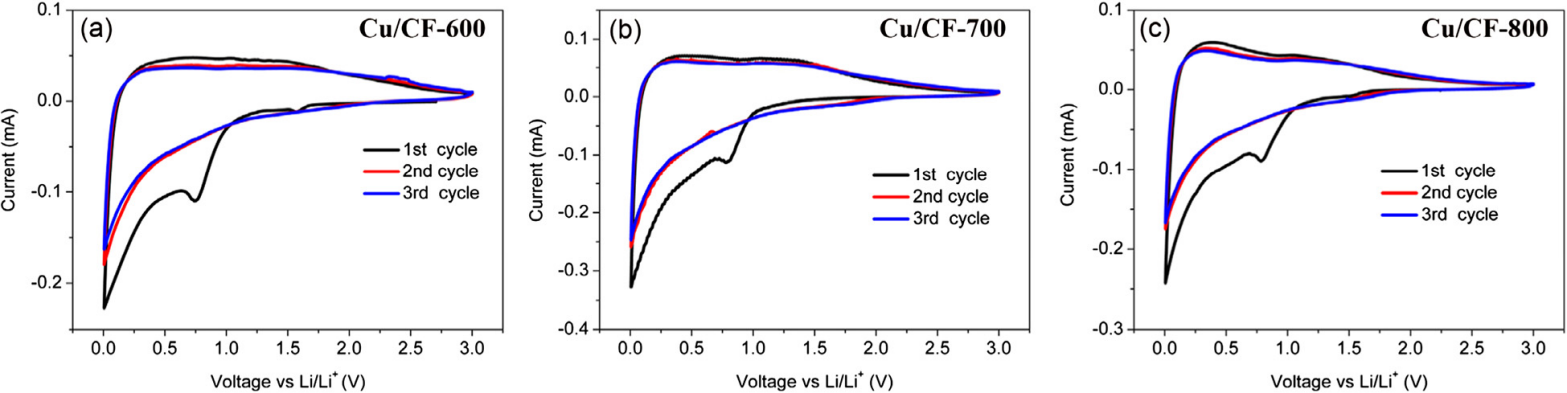
Cyclic voltammograms of first 3 cycles of **a** Cu/CF-600, **b** Cu/CF-700, and **c** Cu/CF-800 samples. The cells cycled between 0.005 and 3 V vs. Li/Li^+^ at scan rate of 0.1 mV s^−1^. In the CV measurements, lithium metal acts as both counter and reference electrode in the two electrode configuration

It is well known that the electrochemical performance is highly dependent on the morphology, crystalline structure, and surface properties. Electrochemical lithium storage properties of Cu/CF were first evaluated by CV measurement and shown in Fig. 4. The Cu/CF electrodes show typical CV curves of the carbonaceous anode materials. In the initial cathodic scans for Cu/CF cells, the irreversible capacity could be indicated by a cathodic peak in the range from 0.73 to 0.79 V, which means electrolyte decomposition in Cu/CF cells. For Cu/CF-800, the irreversible peak is prominent at 0.73 V, which is a little lower than 0.75 V of Cu/CF-700 and 0.79 V of Cu/CF-600 cells. Such difference of peak positions might be due to the residual organic group in the Cu/CF-700 and Cu/CF-600 samples at a relative lower carbonization temperature [36]. In the next 2 cycles, no obvious cathodic peak can be observed at 0.73–0.79 V of Cu/CF cells, which further verifies the electrolyte decomposition and formation of solid electrolyte interface (SEI) completed mostly in the initial discharge. Moreover, the CV curves fitted together very well for the second and third cycles. It implies good reversibility and stability of the Li-intercalation and de-intercalation through electrospun Cu/CF electrode after an initial cycle. Interestingly, anodic scan plots for three Cu/CF electrodes are also different from each other. Cu/CF-600 presents the lowest charging plateau at higher voltage range from 0.5 to 1.0 V, whereas Cu/CF-700 and Cu/CF-800 have prominent peaks below 0.3 V referring to a good de-intercalation mechanism with lithium.

**Fig. 5:**
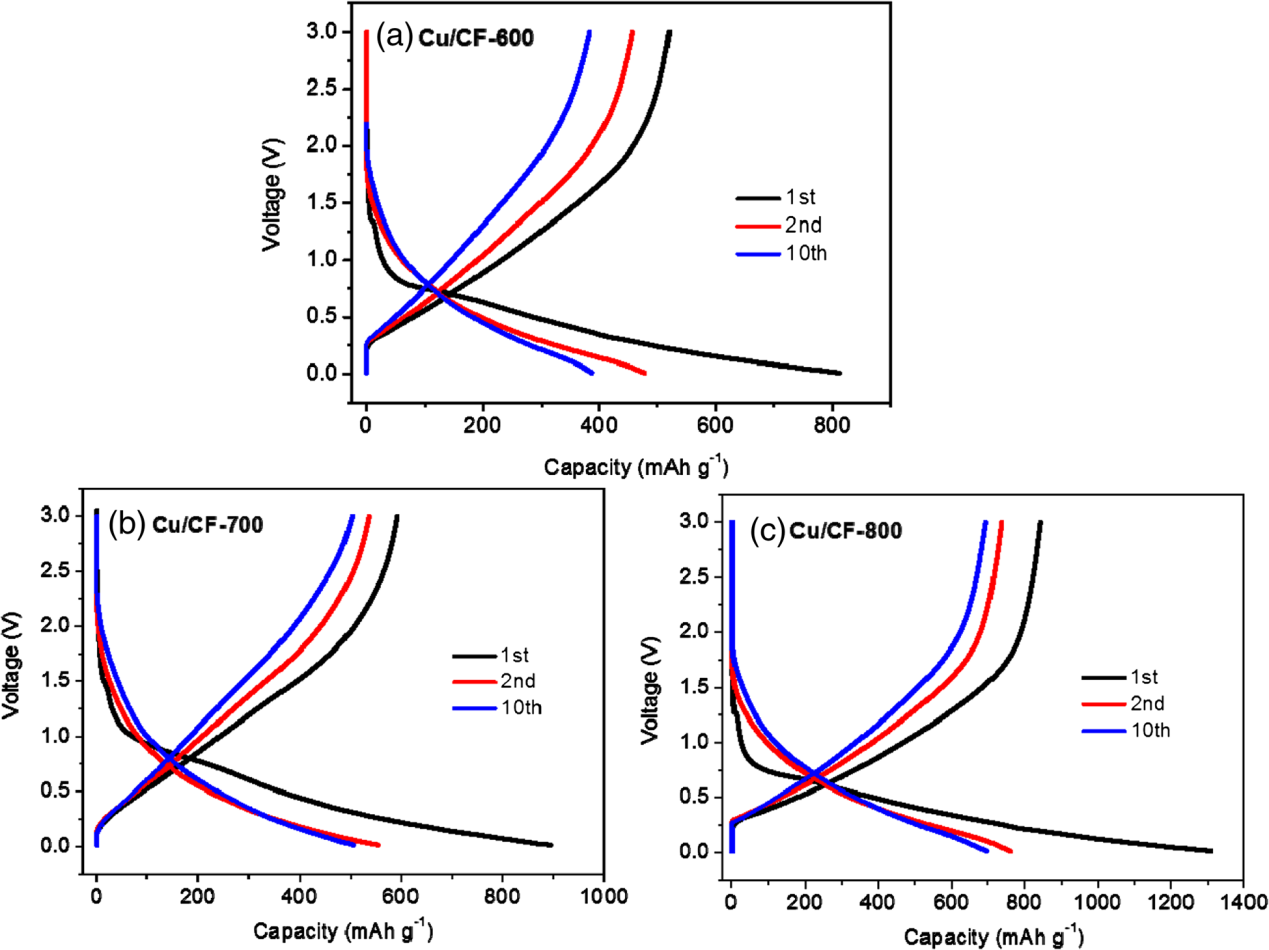
The first, second, and 10th charge/discharge curves of **a** Cu/CF-600, **b** Cu/CF-700, and **c** Cu/CF-800 electrodes vs. Li/Li^+^ at a current density of 100 mA g^−1^ in the voltage range of 0.005–3 V

Shown in Fig. 5 are the charge/discharge profiles from the first cycle to the 10th cycle for Cu/CF-600, Cu/CF-700, and Cu/CF-800 anodes. The initial gentle discharge profiles below 0.8 V of Cu/CF half-cells are due to the formation of SEI film on the surface of anodic electrode [36, 37]. The sloping plateau at ~0.8 V is similar to that of graphite anode [37], suggesting Cu/CF evolves graphitic structures, just as verified by characterizations of XRD and Raman. The Cu/CF-600, Cu/CF-700, and Cu/CF-800 half-cells are first discharged and showed the initial discharge capacity of 811.5, 893.8, and 1308.6 mAh g^−1^, respectively. The initial coulomb efficiencies of Cu/CF-600, Cu/CF-700, and Cu/CF-800 are 64.1, 66.3, and 64.5 %, respectively, and reach almost 100 % coulombic efficiency from the second cycle. The second reversible capacities (477.7 mAh g^−1^ for Cu/CF-600, 554.7 mAh g^−1^ for Cu/CF-700, and 759.8 mAh g^−1^ for Cu/CF-800) are much higher than the theoretical one of graphite anode (372 mAh g^−1^), which is higher than those of the typical carbon nanofiber/metal anodes reported in the literatures (see Additional file 1: Table S1 in the revised support information) [38–42]. Such ultrahigh capacity for Cu/CF might be caused by their special 1D carbonous structure and high-level Cu implanted. For comparison, we prepare pure CF without Cu nanoparticles by the same way to investigate the contribution of CF to the capacity. The first, second, and 10th charge/discharge profiles for CF-600, CF-700, and CF-800 anodes are shown in Additional file 1: Figure S2. The discharge capacities of initial, second, and 10th for CF-600 anode are 566.1, 345.9, and 297.0 mAh g^−1^, respectively, while for CF-700 are 800.5, 435.9, and 318.2 mAh g^−1^; for CF-800 are 905.3, 497.5, and 396.6 mAh g^−1^. From Additional file 1: Figure S2 and Fig. 5, the 800 °C calcined samples possess the highest discharge capacities, regardless of the incorporation of Cu nanoparticles, which is due to the best graphitization at high calcination temperature. Furthermore, it is obvious that Cu/CF owns a superior reversible capacity and better cyclic stability than pure CF at each corresponding temperature. This result demonstrates that the Cu nanoparticles implanted in CF can create more reversible Li^+^ intercalation space, which can significantly increase the reversible capacity of the composite.

**Fig. 6:**
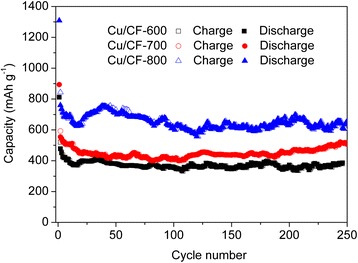
The cycling performances of Cu/CF-600, Cu/CF-700, and Cu/CF-800 at a constant current density of 100 mA g^−1^

The long-term cycling performance of Cu/CF films electrodes was evaluated at 100 mA g^−1^ in the voltage range of 0.005–3 V for 250 cycles. As shown in Fig. 6, all the Cu/CF anodes from different carbonization temperatures show excellent cyclability. The Cu/CF-800 film electrode has the highest reversible capacity (~650 mAh g^−1^). Though the initial coulombic efficiency of Cu/CF-800 is only 64.5 %, it increases dramatically upon cycling, reaching nearly 100 % during the subsequent cycles.

**Fig. 7:**
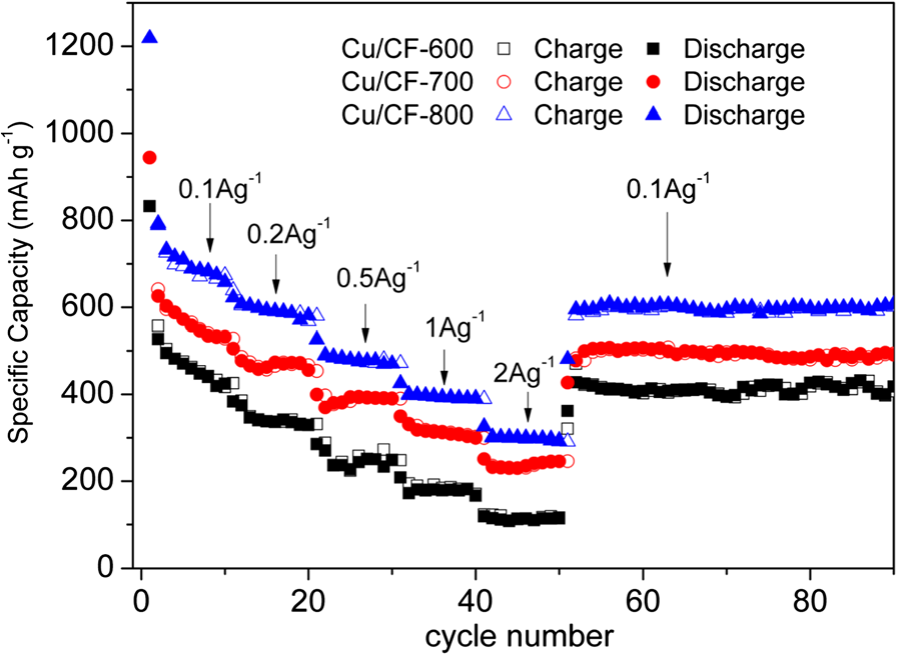
Cycling performances of Cu/CF-600, Cu/CF-700, and Cu/CF-800 anodes under various charge/discharge current densities from 0.1 to 2 A g^−1^

Figure 7 depicts the charge/discharge capability of Cu/CF film electrode (vs. Li) at 0.1 to 2 mA g^−1^. Notably, excellent high-rate performance was observed for the Cu/CF-800 film anode. The reversible capacities are 680.4, 600.7, 477.0, 391.6, and 300.5 mAh g^−1^ at 0.1, 0.2, 0.5, 1, and 2 A g^−1^, respectively. At the current density of 2 A g^−1^ (corresponding to about six C rates), the reversible capacity reaches 35.9 % of the capacity of 0.1 A g^−1^ and is about 80.8 % of the theoretical capacity of graphite. The rate capacities to cycling performance of Cu/CF film anodes are shown in Fig. 7, which display excellent cycling performance with each rate. After a total cycling number of 50 at various current densities between 0.1 and 2 A g^−1^, the specific discharge capacity of Cu/CF-800 film anode can be recovered to 605.8 mAh g^−1^ at 0.1 A g^−1^, with capacity retention of 89.0 %.

**Fig. 8:**
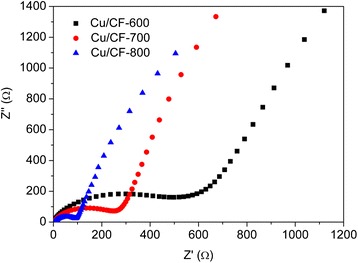
Complex impedance plots of Cu/CF-600, Cu/CF-700, and Cu/CF-800 anodes at the state of discharging (0.5 V)

It is known that the electrochemical performance is highly influenced by the interfacial charge transfer process and the lithium ion diffusion in active materials. To get further insight into the kinetics evolution during charge/discharge cycles, EIS of the carbonized Cu/CF electrodes after 10 cycles at 100 mA g^−1^ were measured and are compared in Fig. 8. As shown in Fig. 8, all the plots contain a depressed semicircle in the high- and medium-frequency regions and an inclined line in the low-frequency zone. The semicircle can be usually assigned to the combination of a solid/electrolyte interface film resistance and charge transfer impedance at the electrode surface, while the line is designated to the Warburg impedance reflecting the solid state diffusion of Li into the bulk of the active materials. Due to the fact that prepared Cu/CF films were placed on current collector directly to be used as the anode, the apparent resistance can be found for the interface between Cu/CF films and current collector, indicating good electronic conductivity [43]. The Cu/CF-800 with the smallest high-frequency semicircle possesses obviously the lowest interface resistance and surface charge transfer resistance, as compared to that of the others, which means faster Li intercalation kinetics and will improve its electrochemical performance [44].

## Conclusions

In summary, free-standing Cu nanoparticle-implanted carbon fiber electrodes have been successfully fabricated via electrospun and calcination techniques. The flexible non-woven Cu/CF composites have smooth, regular, and long fibrous morphologies with Cu nanoparticles percolating throughout the carbon matrix and show stable and high reversible capacity, together with remarkable rate and cycling capabilities as free-standing anodes in Li-ion batteries; especially, the Cu/CF sample calcined at 800 °C (Cu/CF-800) shows the highest charge/discharge capacities, long-term stable cycling performance, and excellent rate performance. Combining with the unique 1D structure of carbon fibers, the introduction of Cu nanoinclusions enhancing the reversible Li^+^ active intercalation/de-intercalation positions and electronic conductivity is believed to be responsible for these.

## Additional files

Additional file 1: Figure S1–Figure S2 and **Table S1.** **Figure S1.** SEM image of (a) electrospinning Cu(NO_3_)_2_/PAN fibers and (b) the amplified image. **Figure S2.** The 1^st^, 2^nd^ and 10^th^ charge/discharge curves of (a) CF-600, (b) CF-700 and (c) CF-800 electrodes vs. Li at a current density of 100 mA g^−1^ in the voltage range of 0.005-3 V. **Table S1.** The comparison of LIBs performance of some typical carbon nanofiber/metal in the literature. (DOC 823 kb)
