# Supplementary material for: Copper Nanoparticle-Incorporated Carbon Fibers as Free-Standing Anodes for Lithium-Ion Batteries
Source: Nanoscale Res Lett. 2016 Mar 31;11:172. doi: 10.1186/s11671-016-1389-6 (PMC4816945; doi:10.1186/s11671-016-1389-6)
Supplement: Additional file 1: Figure S1–Figure S2 — and Table S1. Figure S1. SEM image of (a) electrospinning Cu(NO3)2/PAN fibers and (b) the amplified image. Figure S2. The 1st, 2nd and 10th charge/discharge curves of (a) CF-600, (b) CF-700 and (c) CF-800 electrodes vs. Li at a current density of 100 mA g−1 in the voltage range of 0.005-3 V. Table S1. The comparison of LIBs performance of some typical carbon nanofiber/metal in the literature. (DOC 823 kb) [file 11671_2016_1389_MOESM1_ESM.doc]

**Copper nanoparticles-incorporated** **carbon fibers as free-standing anodes for lithium-ion batteries**

*Pan Han^a,b^, Tao Yuan ^a^, Long Yao^a,b^, Zhuo Han ^a^, Junhe Yang ^a^* and Shiyou Zheng^a^**

^a^ School of Materials Science and Engineering, University of Shanghai for Science and Technology, Shanghai 200093, China.

^b^ School of Environment and Architecture, University of Shanghai for Science and Technology, Shanghai 200093, China.

*Corresponding authors: Phone : +86-21-5527-0305

E-mail: jhyang@usst.edu.cn; syzheng@usst.edu.cn


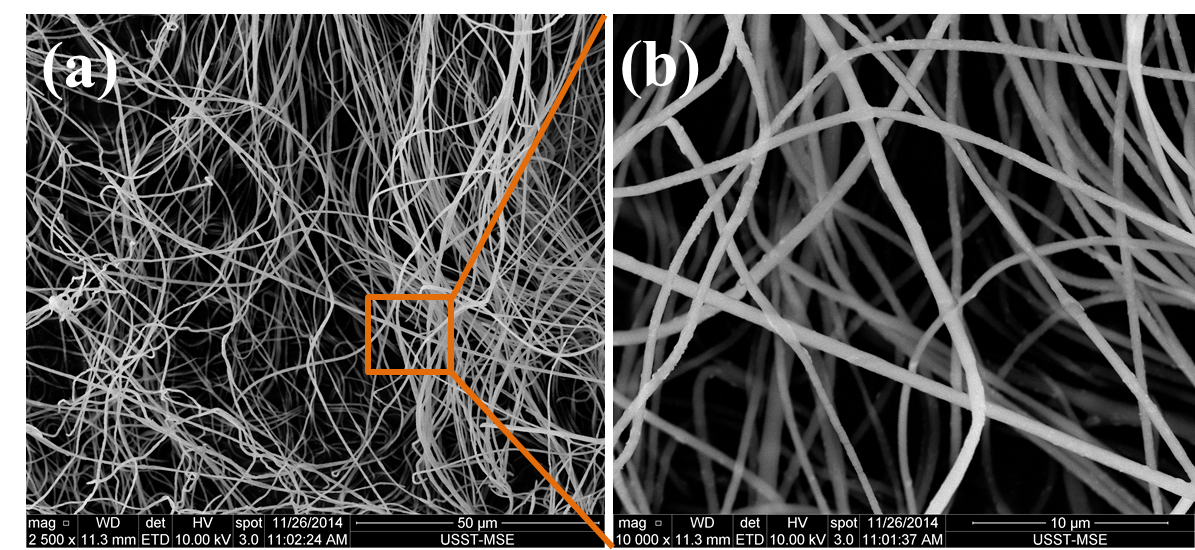


**Figure S1** SEM image of (a) electrospinning Cu(NO_3_)_2_/PAN fibers and (b) the amplified image.


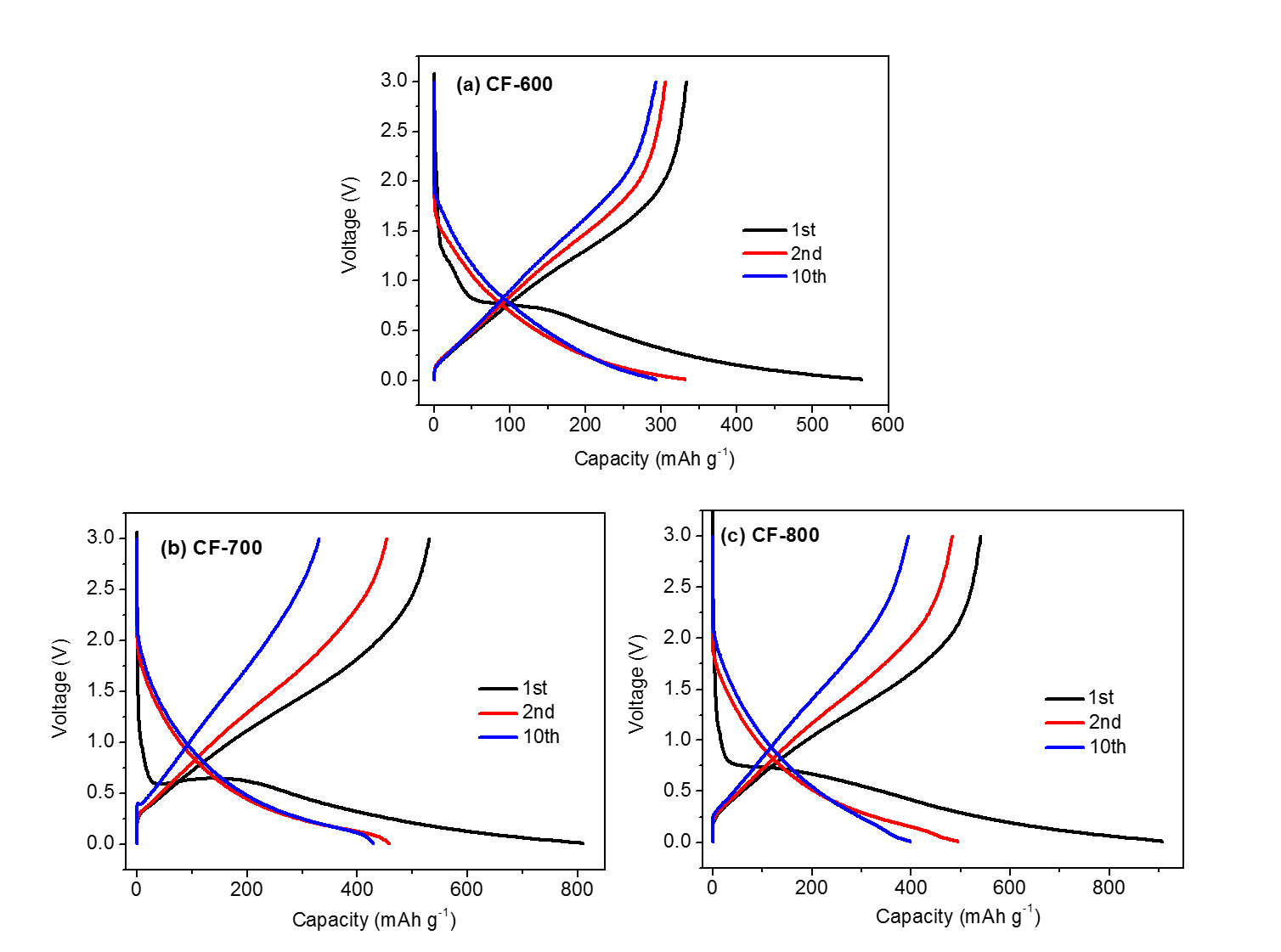


**Figure S2** The 1^st^, 2^nd^ and 10^th^ charge/discharge curves of (a) CF-600, (b) CF-700 and (c) CF-800 electrodes vs. Li at a current density of 100 mA g^-1^ in the voltage range of 0.005-3 V.

**Table S1**. The comparison of LIBs performance of some typical carbon nanofiber/metal in the literature

| **Material** | **Current density（mA g^-1^）** | **Cycle number** | **Specific capacity (mAh g^-1^)** | **Reference** |
| --- | --- | --- | --- | --- |
| **CNFs** | **100** | **100** | **380** | **38** |
| **CNFs** | **100** | **550** | **460** | **39** |
| **CNFs + Sn NPs** | **100** | **20** | **400** | **40** |
| **CNFs + Ni NPs** | **100** | **50** | **500** | **41** |
| **Cu/CF** | **100** | **250** | **800** | **This work** |

CNFs: Carbon Nanofibers, NPs: Nanoparticles.
